# The Crystallization of Amorphous Calcium Carbonate is Kinetically Governed by Ion Impurities and Water

**DOI:** 10.1002/advs.201701000

**Published:** 2018-02-14

**Authors:** Marie Albéric, Luca Bertinetti, Zhaoyong Zou, Peter Fratzl, Wouter Habraken, Yael Politi

**Affiliations:** ^1^ Max‐Planck Institute of Colloids and Interfaces Potsdam‐Golm 14476 Germany

**Keywords:** amorphous calcium carbonate, calorimetry, hydration, X‐ray total scattering

## Abstract

Many organisms use amorphous calcium carbonate (ACC) and control its stability by various additives and water; however, the underlying mechanisms are yet unclear. Here, the effect of water and inorganic additives commonly found in biology on the dynamics of the structure of ACC during crystallization and on the energetics of this process is studied. Total X‐ray scattering and pair distribution function analysis show that the short‐ and medium‐range order of all studied ACC samples are similar; however, the use of in situ methodologies allow the observation of small structural modifications that are otherwise easily overlooked. Isothermal calorimetric coupled with microgravimetric measurements show that the presence of Mg^2+^ and of PO_4_^3−^ in ACC retards the crystallization whereas increased water content accelerates the transformation. The enthalpy of ACC with respect to calcite appears, however, independent of the additive concentration but decreases with water content. Surprisingly, the enthalpic contribution of water is compensated for by an equal and opposite entropic term leading to a net independence of ACC thermodynamic stability on its hydration level. Together, these results point toward a kinetic stabilization effect of inorganic additives and water, and may contribute to the understanding of the biological control of mineral stability.

## 1 Introduction

Since the discovery of the central role of amorphous calcium carbonate (ACC) in the crystallization pathways of biogenic calcium carbonate in sea urchin larva,^1^ the use of amorphous precursors has been recognized as a common strategy employed by many organisms across various phyla to build biominerals with superior properties.^2^, ^3^, ^4^, ^5^ This strategy has also inspired the development of new synthetic routes for controlling morphology, phase, and physical properties of various materials.^6^, ^7^, ^8^

Although unstable under ambient conditions, ACC is surprisingly widespread in biology either as a precursor phase (“transient ACC”), which later transforms into a more crystalline form (calcite or aragonite) or as a stabilized phase (“stable ACC”) sometimes coexisting with crystalline polymorphs.^9^, ^10^ Biogenic ACCs are known to be stabilized by organic, and inorganic additives and when confined in space.^9^, ^11^, ^12^, ^13^, ^14^, ^15^ However, despite extensive research, it is still not fully understood how they are stabilized and what are the mechanisms that control their crystallization.^9^ The “structure” of ACC has been investigated as it is expected to affect its stabilization and exhibits short‐ and medium‐range order (SRO and MRO) up to 10 Å.^16^, ^17^, ^18^ Species‐specific SRO (up to 4 Å) around the Ca^2+^‐ion were identified using Ca K‐edge X‐ray absorption spectroscopy^11^, ^19^, ^20^, ^21^, ^22^ in different biogenic ACCs and a correlation was found between their SRO and their stability. Moreover, anhydrous transient ACCs showed similar short‐range structure to that of the final polymorph.^22^, ^23^ Alternatively, it was also suggested that the phase identified as prestructured ACC is in fact poorly ordered calcite nanocrystals.^24^, ^25^ In sea urchin larval spicules,^26^, ^27^ as well as in gastropod shell nacre,^28^ a transformation mechanism from hydrated ACC via a short‐lived anhydrous ACC to the final polymorph was suggested where the crystalline phase propagates via secondary nucleation from one domain to its neighbor. The energetics of this process was later deduced as a favorable downhill sequence by calorimetric analysis of both biogenic and synthetic ACCs.^29^ Secondary nucleation of ACC nanoparticles were also observed in vitro,^26^, ^30^ where the ACC precursor was transiently stabilized by organic and inorganic additives. Gal et al.^30^ suggested a crystallization mechanism by particle accretion involving partial dissolution–re‐precipitation and solid‐state rearrangement. This process is one case of crystallization by particle attachment mechanisms recently reviewed by De Yoreo et al.^10^

The heterogeneity of biogenic minerals introduces difficulties in interpreting the mechanistic details of the stabilization and transformation of ACC, specifically when the precursor phases and the end products are intermixed at a nanometric scale. Therefore, synthetic ACC has been extensively studied as a model system.^18^, ^29^, ^31^, ^32^, ^33^, ^34^, ^35^, ^36^ As their biogenic counterparts, synthetic ACCs present S/MRO up to 10 Å that are different^17^, ^33^, ^34^, ^35^ or similar^33^, ^37^ to the one of the crystalline phases of CaCO_3_. In addition, for structurally identical ACCs, physical parameters such as particle size affect their stability and transformation pathways.^32^ Nevertheless, whether ACC stabilization is kinetic or thermodynamic and what are the mechanisms leading to its crystallization still highly debated. Moreover, besides the pioneering study conducted by Radha et al.^29^ experimental isothermal calorimetric data of ACC is limited.

In order to better understand the mechanisms of ACC stabilization and crystallization, the effects of the different additives on the energetics, on the local structure and, importantly, its evolution during crystallization need to be determined. Here, we address these questions by (1) following the structural rearrangement accompanying dehydration and crystallization of synthetic ACCs using in situ total X‐ray scattering and (2) establishing the energetic landscapes by gravimetric analysis coupled with isothermal or differential scanning calorimetry. We consider ACCs with and without inorganic additives commonly found in biogenic ACC, namely magnesium and phosphate ions, with different water content and particle size to distinguish the effects of each of these physicochemical parameters.

## 2 Results

### 2.1 Compositional, Morphological, and Structural Characterization

The synthesized ACCs exhibit the typical morphology of aggregated spheres with an average diameter of 65, 75, and 39 nm for ACC, Mg‐ACC and P‐ACC, respectively (**Table** **1**, Figure S1, Supporting Information). Relative to ACC without additives, the addition of PO_4_^3−^ leads to the formation of ACC with smaller particles and a narrower size distribution, whereas the addition of Mg ions did not affect the mean particle size but increased the size distribution. The final composition of each of the precipitates was determined using inductively coupled plasma/optical emission spectrometry (ICP‐OES) and thermogravimetric analysis (TGA) measurements (Table 1). The obtained materials contain between 1.3 and 1.4 H_2_O molecules per CaCO_3_ and trace amounts of Na ions. The water considered here refers mainly to bulk water, whereas surface water was estimated to comprise up to 2% of the total water (see Text S1 in the Supporting Information). We note that a small amount of hydroxyl ions may be present in Mg‐ACC samples.^17^

**Table 1:** Chemical composition (mol. ratio), mean particle size (nm), and standard deviation (sdv) of the distribution (see the Supporting Information for the size distribution) of the synthetized ACC samples used in the heating experiment (X‐ACC) and in the isothermal experiments (X‐ACC_iso_). The chemical composition of the ACC samples was determined by ICP‐OES. Water content was determined from the weight loss in TGA measurements. CO_3_^2−^ was calculated using mass and charge balances by assuming charge neutrality for the amorphous precipitate

Sample name	Ca^2+^	Na^+^	Mg^2+^	CO_3_^2−^	PO_4_^3−^	H_2_O	Mean particle size	Sdv of the size distribution
ACC	100	0.7	0	100.3	0	140	65	44
Mg‐ACC	100	1.0	2.4	103.1	0	140	75	47
P‐ACC	100	1.0	0	90.3	6.8	130	39	21
ACC_iso_	100	3.2	0	101.6	0	100	52	27
Mg‐ACC_iso_	100	1.0	4.8	105.3	0	100	52	26
P‐ACC_iso_	100	2.2	0	96.7	2.9	100	45	22

The X‐ray scattering profiles of all prepared ACCs are similar with two distinct broad peaks at around *q* = 22 nm^−1^ and *q* = 31 nm^−1^, a broad hump at 54 nm^−1^ and a shoulder at 62 nm^−1^ (Figure S2, Supporting Information) in agreement with previous reports.^38^ The different intensities at low *q* values (1–1.6 nm^−1^) might reflect the presence of different inhomogeneities within the samples (sample packing, small pores, clusters with different scattering power etc.). The pair distribution functions (PDF), *G*(*r*) of the three hydrated ACC samples (with and without additives) calculated from the structure functions, *S*(*q*), exhibit clear short‐ to medium‐range order extending up to 8–10 Å (Figure S3, Supporting Information). The positions of the main maxima of all PDFs are around 2.4, 2.9, 4.1, and 6.2 Å. No significant differences could be evidenced between the samples, except for a slight broadening of the peak around 4.1 Å for P‐ACC. Using the total and partial PDFs of monohydrocalcite, vaterite, calcite, and ACC from simulation and experimental data reported in refs. 17, 39, 40, 41 (Table S1, Supporting Information), we assigned the distances as follows: the peak at 2.4 Å contains contributions mainly from Ca–O pairs, the peak at 2.9 Å from O–O pairs, the peak at 4.1 Å from Ca–O and Ca–Ca pairs (1st shell) and the peak at 6.2 Å mainly from 2nd shell Ca–Ca pairs.

### 2.2 Heat Induced Transformation of the Studied ACCs

**Figure 1:**
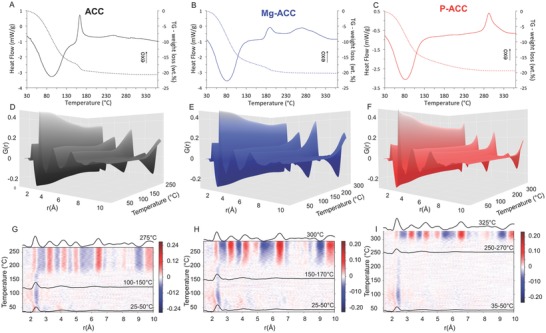
TGA–DSC analysis of A) ACC, B) Mg‐ACC, and C) P‐ACC, PDF from in situ heating XRD measurements of D) ACC, E) Mg‐ACC, and F) P‐ACC and differential dPDF/d*T* maps of G) ACC, H) Mg‐ACC, and I) P‐ACC, averaged PDFs at selected temperatures are superimposed on the maps (black curves). For ACC, averaged PDFs between 25 and 50 °C (1.4 H_2_O), between 100 and 150 °C (0.4 H_2_O) and at 275 °C. For Mg‐ACC, averaged PDFs between 25 and 50 °C (1.4 H_2_O), between 150 and 170 °C (0.2 H_2_O) and at 300 °C. For P‐ACC, averaged PDFs between 35 and 50 °C (1.3 H_2_O), between 250 and 270 °C (0.4 H_2_O) and at 325 °C.

The transformation of ACC upon heating was monitored using TGA/differential scanning calorimetry (DSC) and in situ heating total X‐ray scattering measurements (**Figure** **1**). All samples show an initial weight loss concomitant with an endothermic event around 80 °C as described before^41^, ^42^ (Figure 1A,B). This water loss event removes about 75–80 wt% of total water, and occurs at similar temperatures for all studied ACCs. The remaining water in ACC and Mg‐ACC is expelled during crystallization, whereas in P‐ACC it is expelled gradually. The crystallization event is recognized by an exothermic peak in the DSC curves. The onset‐, the peak‐temperature, and the shape of the exothermic peak differ from sample to sample. The main crystallization peak of pure ACC is observed around 160 °C with a small additional bump at around 245 °C. Previous thermal analysis results showed dependence of the crystallization temperature on the particle size with smaller particle size exhibiting higher crystallization onset temperatures.^32^ In accordance, the observed crystallization peaks are assumed to be the result of the ACC particle size distribution.^32^ In the presence of Mg^2+^ and PO_4_^3−^ ions the crystallization temperature increases relative to ACC without additives (Figure 1B,C). For Mg‐ACC, two exothermic events can be observed at 187 and 270 °C. Also in this case, the second peak could be due to the transformation of very small particles, which can be seen in the SEM images (Figure S1, Supporting Information). Another possibility for the observation of two crystallization events could be an inhomogeneous distribution of the Mg ions within the Mg‐ACC sample.^43^ In this case, Mg‐rich ACC particles will crystallize at higher temperature relative to Mg‐poor particles.^18^ However, based on the thermal behavior of the Mg‐ACC alone we cannot determine if this phase separation occurs during the synthesis of ACC or if it occurs just during heat treatment. Nevertheless, the onset of the first crystallization peak is shifted to higher temperature compared to the one of pure ACC, suggesting that at least part of the Mg is homogenously incorporated in ACC. In the case of the P‐ACC, the transformation occurs at around 290 °C. Integration of the exothermic peaks of each sample after correction of the endothermic contribution of H_2_O evaporation for ACC and Mg‐ACC due to the minor water loss, reveals that the enthalpy of crystallization for ACC is −13 kJ mol^−1^ at 160 °C, for Mg‐ACC −12 kJ mol^−1^ between 187 and 270 °C and for P‐ACC −9 kJ mol^−1^ at 290 °C. Calcite was the only crystalline phase found in all cases, however a phosphate‐rich amorphous phase (ACP) remained untransformed for P‐ACC. Assuming no chemical reaction during crystallization and that ACP is carbonate‐free, the Ca:P in ACP can be assessed to be around 1.4. Therefore, incomplete crystallization would account for about 10% reduction of the crystallization enthalpy. The observed 30% reduction suggests either an enthalpic contribution of phosphate ions to ACC stability or that others processes such as phase separation take place at the same temperature.

To describe the structural changes upon dehydration and crystallization, we acquired the X‐ray scattering patterns of the three ACC samples during in situ heating and calculated the PDF at every temperature (Figure 1D–F). For all types of ACC the intensity of the 2.4 Å peak decreases for temperatures up to 120 °C, while the intensity of all other peaks remain similar. Although we cannot quantitatively determine the coordination numbers due to the lack of density data for these phases at each temperature, this decrease might suggest a reduction in the total Ca–O coordination number prior to crystallization, in agreement with MD simulations.^40^

The differential of the PDF data set as a function of temperature (dPDF/d*T*) (Figure 1G–I) allow a better visualization of the structural changes upon heating. The color scale indicates the rate of the intensity change of the PDF profiles with temperatures. In this representation, the appearance of adjacent blue and red vertical “stripes” designates a peak shift upon heating. As a reference for the peak positions, three calculated PDFs at selected temperatures are superimposed on each map (Figure. 1G–I): the PDF of the initial ACC phases, the PDF before and the one after the crystallization (also see Figure S4, Supporting Information). The differential maps demonstrate that structural rearrangements occur during the major water loss event between 25–120 °C, in a limited radial range (≈5–6 Å for ACC, 6–7 Å for Mg‐ACC, and 4–5 Å for P‐ACC). In particular, the peak at 2.4 Å assigned to the Ca–O shifts to lower distances for all ACCs. This shift originates from an intensity decrease around 2.5 Å accompanied with an increase around 2.2 Å. The peak at 4.1 Å (Ca–O_carb_ and Ca–Ca pairs) shifts to lower r for ACC and for Mg‐ACC. This shift occurs at *T* < 65 °C for ACC and at 65 °C < *T* < 85 °C for Mg‐ACC. For P‐ACC, this peak does not change at an appreciable rate. Other small variations observed at distances up to 7 Å in ACC and in Mg‐ACC are hard to interpret due to the low signal/noise. From *T* = 120 °C until the onset of the crystallization, the rate of change of the peak at 2.4 Å decreases for all the materials, and all other peaks remain unchanged within the sensitivity of the measurement.

### 2.3 Humidity Induced Transformation

We used an innovative approach based on combined gas phase isothermal calorimetry and microgravimetry (see the Experimental Section) to accurately measure the transformation's dynamics of ACC in hydrated environment. We considered a large set of samples with different particle size, composition and water content in order to determine which of these physicochemical parameters is most important for the crystallization kinetics and thermodynamics.

When the ACC particle size is the only variable parameter (ACC without additives and with the same water content), a slight variation in the onset time of crystallization (small particle sizes delay crystallization) is observed but without obvious differences in the heat‐flow evolution (Figure S5, Supporting Information). This delay in crystallization could be understood in light of recent results^32^ that reported that smaller ACC particles are more thermally stable than larger ones. In that work^32^ it was suggested that the molecular moieties near the surface could rearrange more easily in an amorphous particle (thereby lowering the interface energy with air) relative to a crystalline solid.

**Figure 2:**
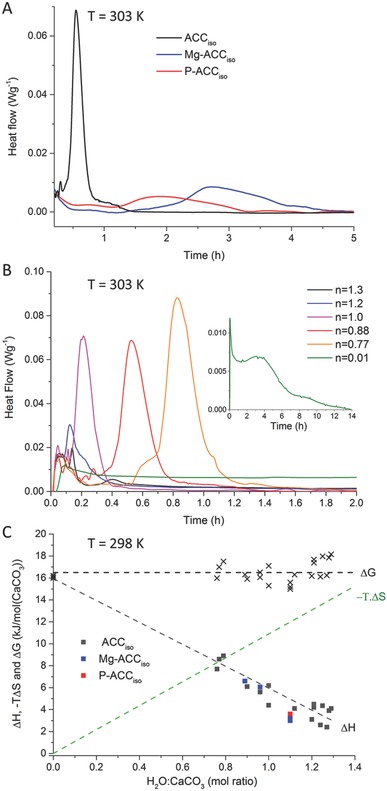
A) Heat flow evolution during crystallization induced by humidity (at 30 °C, i.e., 303.15 K) of ACC_iso_·1.H_2_0 (45 nm), Mg‐ACC_iso_·1.H_2_0 (2.5% Mg, 45 nm), and P‐ACC_iso_·1.H_2_0 (3%P, 40 nm), B) Isothermal calorimetry measurements of differently hydrated ACC·*n*H_2_O samples without additives and containing *n* = 0.01, 0.77, 0.88, 1, 1.2, and 1.3 H_2_O (at 30 °C, i.e., 303.15 K) and C) Δ*H* (ACC, Mg‐ACC and P‐ACC are represented respectively by grey, blue, and red squares), −*T*∙Δ*S*(with −*T*∙dΔ*S*/d*n*_H2O_ = 10.9 kJ mol^−1^ for *T* = 298K, see Text S2 in the Supporting Information for calculation green dashed line) and Δ*G* (Δ*G* = Δ*H* – *T*∙Δ*S*, dark crosses and dashed dark line) of ACC with different hydration levels with respect to calcite, with *T* = 25 °C, i.e., 298 K.

With respect to additives (ACCs with the same water content and similar particle size, Table 1, ACC_iso_), even in low concentrations, both Mg^2+^ and PO_4_^3−^ have a stabilizing effect and reduce the crystallization rate as seen in the delay of the crystallization starting point as well as in the increase of the time needed for its completion (**Figure** **2**A). For ACC containing 6.8 mol% PO_4_^3−^, the transformation rate was so reduced that the heat‐flow could not be determined unequivocally due to the sensitivity limit of the technique. We therefore used ACC with lower phosphate content (Table 1, P‐ACC_iso_).

The effect of water on the transformation was studied by comparing the isothermal measurements for several additive‐free ACCs with the same particle size but different amount of water (CaCO_3_∙*n*H_2_O with *n* = 0.77–1.3). These samples were obtained by storing similar ACC samples under vacuum for several days. One sample was further dehydrated (*n* = 0.01) by heat treatment (200 °C for 2 h). The results show a strong correlation between the hydration level of ACC and the crystallization rates (Figure 2B).

In addition to kinetics, the experimental setup allows measuring the enthalpy of crystallization at 30 °C, Δ _c_ *H* ^30^. Interestingly, whereas the crystallization kinetics was found to be strongly dependent on the presence of additives, Δ_c_ *H* ^30^ was similar for the three samples (ACC_iso_, Mg‐ACC_iso_, and P‐ACC_iso_) lying between −4 and −6 kJ mol^−1^ (Figure 2A). However, as can be seen in Figure 2C, the enthalpy of ACC relative to calcite Δ*H* (= −Δ _c_ *H* ^30^) decreased linearly with hydration level. From these data, we measured the partial molar enthalpy of dehydration of ACC (i.e., dΔ*H*/d*n*_H2O_, where *n*_H2O_ is the number of water moles) to be −10.3 ± 0.6 kJ mol^−1^. Thus, anhydrous ACCs are kinetically stabilized relative to hydrated ACCs, and the molar enthalpy of ACC relative to calcite is lower for hydrated ACCs than for anhydrous ACC (Δ*H*_(hydrated ACC)_ < Δ*H*_(anhydrous ACC)_).

Finally, to investigate the structural rearrangement of ACC in a hydrated environment we exposed the ACC samples to high relative humidity (RH = 85%) and followed their crystallization in situ using X‐ray total scattering. The differential maps (dPDF/d*T*) are depicted in Figure S6 in the Supporting Information. Under constant high relative humidity, we observe simultaneous peak appearance in the entire r‐range at the same positions as those of the final polymorph and their intensity increases with time. This indicates a constant formation of the crystalline end product, without a noticeable intermediate stage, in agreement with previous reports.^35^ The crystalline products after 1 h were a mixture of calcite and vaterite for ACC, and P‐ACC (which also contained a small amount of phosphate rich amorphous phase), and Mg‐calcite for Mg‐ACC. The onset of crystallization and the time required for its completion varied significantly between the different types of ACCs, with Mg‐ACC and the P‐ACC showing dramatically slower kinetics.

## 3 Discussion

### 3.1 Structural Rearrangement upon Dehydration

The structure of hydrated (additive free) ACC revealed by our X‐ray PDF experiments is similar to that reported in the literature for several other ACC types, with well‐defined SRO and MRO up to 8–10 Å^16^, ^17^, ^18^, ^19^, ^41^ dissimilar to any crystalline polymorphs. In agreement with previous reports^41^ we observed only small SRO and MRO changes in the total PDF between hydrated and (thermally) dehydrated ACC. However, by following the dehydration process in situ, we demonstrated that structural rearrangements do occur before *T* = 120 °C, during the main dehydration step in which the majority of the water molecules are expelled (at 120 °C the hydration level is a 0.4:1 water/Ca). At this temperature range both SRO and MRO are affected by the water loss. From *T* = 120 °C and up to the onset of the crystallization, the removal of remaining water only affects the first Ca–O shell with no evident change of the MRO. These observations are consistent with water being an integral structural component of the first ACC coordination shells^36^, ^39^ rather than being separated into water–carbonate rich channels or pores.^15^, ^16^

### 3.2 Influence of Additives on ACC Structure

We use low concentration of additives (Mg^2+^, PO_4_^3−^) such that the PDF is dominated by the scattering from calcium and carbonate (O, C atoms). Our results show that within our experimental resolution and sensitivity, the SRO and MRO of ACC is little affected if at all by the presence of the additives. At room temperature, the structure of ACC containing Mg (2.4% Mg:Ca) is the same as the one of pure ACC in agreement with previous studies.^18^, ^44^ According to Radha et al.^18^ the PDFs of Mg‐ACC is significantly affected by the Mg presence only from Mg:Ca ratio of 40% and above. The structure of P‐ACC (≈7% P:Ca) is also very similar to ACC with a slight broadening of the first Ca–Ca shell, consistent with the presence of the larger PO_4_^3−^ ion within this r‐range. This result is in accordance with Kababya et al.,^31^ who demonstrated that at these concentrations phosphates, water and carbonates are homogeneously distributed at the molecular level. We note however, that due to overlap between the contributions from several atomic pairs in the PDF of CaCO_3_, the scattering technique is somewhat less sensitive to small variations in the short‐range structure (at the level of the first coordination spheres) relative to spectroscopic techniques such as nuclear magnetic resonance (NMR) or X‐ray absorption spectroscopy (XAS).^15^, ^45^

### 3.3 Crystallization and Stabilization Mechanisms

#### 3.3.1 Additives

The transformation of ACC by heating or by humidity follows different pathways leading also to different products as already noted before.^32^, ^33^ The solid‐state transformation induced by heating is evidenced in the PDF evolution, which shows structural rearrangement only within a limited length scale (up to 7Å), followed by the appearance of peaks at the onset of crystallization at higher temperatures. As in the hydrated state, a comparison of the anhydrous phases shows only minor structural differences. Such comparison is, however, misleading as it overlooks the dynamics of dehydration. During heat‐induced transformation the additives affect the temperature of the transformation and influence the range of structural changes. Although this is less evident under humidity, where structural reorganization at all length‐scales (SRO to LRO) occurs by local dissolution–precipitation without preceding SRO rearrangement, the time needed to start and complete the transformation is largely affected by the presence of additives. Hence, the dynamics of the transformation seems to largely depend on the presence of additives. As the strength of the interaction with H_2_O depends on the polarizing power and electronic structure of the neighboring ions, it can be envisaged that each additive can affect the kinetics of dehydration (a step required for crystallization) in a different way. Water is expected to bind, at least partially, to Mg^2+^ ions in Mg‐ACC^13^, ^46^ and to both carbonates and phosphates in P‐ACC.^31^ The presence of hydroxyl ions in Mg‐ACC has been suggested.^17^ If present, OH^−^ ions are also expected to bind to Mg and therefore affect the kinetics of the transformation, as dehydroxylation is also a required step before crystallization. Therefore, we propose that the binding between additives and water and/or hydroxyl ions is a key for ACC kinetic stabilization by decreasing ions mobility. This is in line with previous suggestions for the crystallization of amorphous magnesium carbonate to magnesite in which structural rearrangement and Mg^2+^ dehydration were pointed out as kinetic barriers.^18^, ^36^ Moreover, it was shown that during P‐ACC crystallization, phosphate and water diffuse together out of the ACC phase.^31^ Even at the low concentrations of additives studied, both, Mg^2+^ and PO_4_^3−^, increase the temperature of crystallization when induced by heating, and delay the crystallization when induced at high RH. The enthalpy of crystallization determined from isothermal calorimetry appears independent of the presence of additive. This, in addition to the lack of structural differences, suggests that the stability of ACC by additives derives mostly from a kinetic effect.

In addition, the increased kinetic stability in the presence of either additive may result also from different mechanisms. In the presence of magnesium ions, the crystalline product was always calcite, thus the increased stability of ACC may be related to vaterite inhibition, the otherwise kinetically more favorable crystalline form. In the presence of phosphates, the ACC particle size is largely reduced, which can have an additional effect on the thermal stability of the P‐ACC phase.^32^ However, the stabilizing effect of phosphate ions is not limited to reduction of particle size as can be learnt from comparing the isothermal crystallization time between additive free ACC and P‐ACC with similar particle size (≈45 nm) (Figure 2A). The latter showed delayed crystallization under high humidity conditions. As opposed to magnesium ions, phosphates cannot be incorporated in either calcite or vaterite thus a thermodynamic geometric frustration mechanism in which ACC is locked in an unfavorable state has been suggested previously.^14^, ^31^ In addition, the presence of phosphate ions may also contribute an enthalpic stabilization term, which we have not, however, been able to quantify. Nevertheless, especially given the very low phosphate concentration used here, the observed kinetic effect seems to dominate the stabilization mechanism. Finally, the presence of additives in ACC and the end products may also reduce the entropy change of the crystallization, therefore, stabilizing the amorphous phase. This contribution to the reaction free energy is however expected to be small at room temperature.^29^, ^47^

#### 3.3.2 Water

Our results shed new light on the effect of water on the kinetics and the thermodynamic‐stability of ACC. We found anhydrous ACC to be kinetically more stable than hydrated ACC in agreement with Bushuev et al.^39^ Our results show that the intrinsic ACC water content affects both the time needed to start the crystallization (peak onset in Figure 2B) and the amount of time needed for its completion (peak width). This links the water content to (1) possible solubility difference between hydrated and anhydrous ACC which will determine the crystallization onset, and (2) the ions' mobility, which in turn will determine the transformation rate. Indeed, the presence of structural water in hydrated ACC is expected to accelerate the local dissolution/re‐precipitation. Moreover, we established a linear correlation between the enthalpy of crystallization and the amount of water in ACC, with larger enthalpy relative to calcite for anhydrous ACC than for hydrated ACC (with the relation: dΔ*H*/d*n*_H2O_ = −10.3 ± 0.6 kJ mol^−1^). Our results agree with the trend predicted by Bushuev et al.^39^ within the corresponding hydration range. These findings seemingly contrast those of Radha et al.^29^ who reported the opposite trend in which hydrated ACC has a larger enthalpy relative to calcite (Δ*H* = 24 kJ mol^−1^) than anhydrous ACC (Δ*H* = 16 kJ mol^−1^). The reported ACC, however, was synthesized at higher pH levels relative to the ACC used here, which likely result in ACCs with different stabilities.^34^, ^48^ We stress however, that the values for anhydrous ACC obtained by our method are compatible with those obtained by Radha et al.^29^ by isothermal acid solution calorimetry.

The thermodynamic stability of ACC is described by the molar free energy Δ*G*. To estimate it, the molar entropy difference to calcite Δ*S* (= −Δ_c_ *S*, entropy of crystallization) needs to be taken into account. At room temperature, for an amorphous‐to‐crystalline transformation, *T*Δ*S* is expected to be small.^29^ However, if dehydration is involved as in the present case, this term can represent a significant contribution to the free energy of the reaction.^49^ The molar entropy −dΔ*S*/d*n*_H2O_, for the incorporation of water in hydrated crystalline polymorphs of Ca‐ and Mg‐carbonates can be calculated from experimental thermodynamic data^50^, ^51^, ^52^, ^53^ (see Text S2 in the Supporting Information) and seems to be rather independent of the starting crystalline phase.^54^ Using the available data we calculated the entropic contribution –*T*dΔ*S*/d*n*_H2O_ to the molar free energy for the crystallization to be 10.9 kJ mol^−1^ at 25 °C (298 K). Similar results have been obtained by molecular dynamic simulation.^54^ Considering the entropic contribution due to the amorphous‐to‐crystalline transformation to be negligible with respect to the one due to water incorporation, and taking the latter to describe the molar entropy difference to calcite, we obtain a constant molar free energy for the transformation of ACC to calcite, regardless of its water content (Figure 2B). Against our expectations, the counter‐balancing effect of the enthalpy and the entropy contributions results in no thermodynamic preference for any hydration level from 0.01 to 1.3 H_2_O at 25 °C. The compensation is observed when dΔ*H*/d*n*_H2O_ = *T* dΔ*S*/d*n*_H2O_. Considering our experimental error, dΔ*H*/d*n*_H2O_ varies between 9.7 and 10.9 kJ mol^−1^ and therefore this compensation is expected for a temperature range relevant for biological systems (about 0 to 30 °C).

### 3.4 Implications for Biomineralization

Phosphate and magnesium ions are common additives in biological ACC systems but they are employed differently. Phosphates are commonly found in long‐term stabilized ACCs,^14^, ^19^, ^55^ whereas magnesium ions are present in both “stable” and “transient” ACCs.^9^ Our results shed additional light on this distribution. The kinetic inhibition of the crystallization by phosphate ions is strong even at low concentrations. Higher concentrations, as usually found in living organisms (10–20%), can easily prevent ACC from crystallizing for long periods of time.^14^ Moreover, at these concentrations the thermodynamic effects of phosphate addition might play a significant role resulting in a highly stabilized phase.^31^ The lesser kinetic stabilization effect of magnesium ions (note almost double magnesium concentration in Figure 2A) on the other hand, makes it suitable to temporarily stabilize ACC as a precursor phase. In addition, the presence of magnesium ions in ACC in biologically relevant concentrations (i.e., 5%) is sufficient to guide calcite polymorph selection.

A central question in biomineralization, especially when precursor phases are involved, is the determination of the driving forces that govern the crystallization pathways. The transition from hydrated ACC precursor to crystalline phase in sea urchin spicules via an intermediate anhydrous ACC phase was proposed to follow a thermodynamic downhill sequence.^29^ Our work on synthetic ACCs suggests however that hydrated and anhydrous ACC exhibit similar thermodynamic stability, but that the dehydration step introduces a significant kinetic barrier for the crystallization. This is then likely to be the rate‐limiting step of the transformation. In our study, hydrated ACCs stored under vacuum for several days have lost some of their water content without crystallizing into calcite, suggesting that partial dehydration can occur spontaneously under these conditions. Spontaneous dehydration without crystallization has been previously suggested for two biological systems; in both sea urchin larval spicules^26^, ^27^ and in abalone nacre,^28^ hydrated ACC is short lived and transforms into anhydrous ACC, which persists for longer periods of time before crystallizing into calcite and aragonite, respectively.^1^, ^12^ In the case of the sea urchin larval spicule, the transformation propagates through secondary nucleation^26^ originating in an initially deposited calcite crystal.^1^, ^56^ This mechanism can help overcome the kinetic barrier associated with the transformation.^57^

The energy landscape for the transformation largely depends on the pH‐level of the syntheses,^29^, ^48^ however, the pH at which biominerals are formed is in most cases unknown, with the exception of foraminifera biomineralization where pH values around 9 were measured.^58^ Moreover, biological systems are more complex than the systems studied here involving an organic matrix, membrane confinement and complex crystallization mechanisms, including particle accretion, solids‐state transformation and local dissolution and re‐precipitation mechanisms.^10^, ^30^ For example, certain organic molecules also seem to manipulate water mobility and therefore govern ACC stabilization and transformation.^59^ Thus, direct conclusions cannot be drawn from the in vitro to the in vivo systems, however, the boundaries within which biological systems operate may be established from in vitro studies such as this one.

## 4 Conclusion

Our results show that ACC stabilization in the studied systems is dominated by kinetic control and depends on several factors: particle size, the presence of additives and the water content. Using in situ methodology and novel analyses, we describe the crystallization dynamics of ACC, with and without additives induced by heating or high relative humidity. Despite similar initial short‐ and medium‐range order of all studied ACC samples studied, additives increase the thermal stability of the amorphous phase and, at high humidity, delay the onset of crystallization as well as the time required for its completion. Nonetheless, neither additive has a large effect on the crystallization enthalpy at room temperature. Most importantly, water content was found to correlate linearly with the molar enthalpy difference to calcite. Due to a balancing effect of the molar entropy change between hydrated and anhydrous ACC the free energy of ACC relative to calcite is independent of its hydration level from 0 to 1.3 H_2_O. Water, however, has a strong effect on the ion mobility and is the key to structural rearrangement as is reflected in the structural changes occurring during water loss at moderate temperatures. We anticipate that the developed methodology will aid further study, for example to determine to what extent the role of additives is synergistic or antagonistic in the stabilization of ACC. In addition, the present study contributes to the understanding of biological mineralization and may guide materials synthesis for various applications.

## 5 Experimental Section

*ACC Synthesis*: ACC was synthesized in the presence (or absence) of inorganic additives (Mg^2+^, PO_4_^3−^ ions) by mixing of CaCl_2_ (or CaCl_2_/MgCl_2_, ratio: 9/1) and Na_2_CO_3_ (or Na_2_CO_3_/NaH_2_PO_4_, ratio: 9/1) solutions. The mixing was either performed using pipettes on site at the European Synchrotron Research Facility (ESRF) beamline in order to have fresh samples or using a controlled titration set up as in.^32^ The latter was used to control the particle size of ACC. The titration set up allows measuring the pH, which changes from 11.4 to 10.6 from beginning to the end of the reaction. The ACC synthesis was followed by a fast filtration and drying procedure using cold ethanol (4 °C, 100%). The samples were stored for further use in a vacuum desiccator. By varying the concentration of the initial solutions (CaCl_2_ and Na_2_CO_3_) pure ACCs (with no additives) with different particle sizes were also synthetized. Pure ACC with water content of *n* = 0.01 used in isothermal experiment was obtained by heat treatment at 200 °C for 2 h. Pure ACCs with water content from *n* = 0.77 to 1.3 were obtained by storing the ACC under vacuum for several days.

*Chemical Analysis*: The chemical composition of the ACC samples was determined by ICP‐OES (Optima 8000, Perkin Elmer Inc., USA) after dissolution of powdered samples in the acidic solution (167 µL HNO_3_ and 333 µL HCl). Water content was determined from the weight loss in TGA measurements. CO_3_^2−^ was calculated using mass and charge balances by assuming charge neutrality for the amorphous precipitate.

*TGA and DSC*: TGA coupled with DSC measurements were performed using a SENSYS evo TGA‐DSC apparatus (SETARAM Instrumentation, Caluire, France) using aluminum crucible. Weight loss and heat flow were measured during programmed heating (25−500 °C at 1.5 °C min^−1^), under a stream of gas consisting of a mixture of 20 mL min^−1^ nitrogen and 10 mL min^−1^ oxygen. Weight corrections for flow unbalances were made in order to accurately subtract the background and obtain reliable quantitative data for weight loss and heat flow.

*Microgravimetry and Isothermal Calorimetry*: The enthalpy change associated with the ACCs crystallization was measured isothermally at *T* = 303 K by means of a SENSYS evo apparatus coupled with a Wetsys humidity generator (SETARAM Instrumentation, Caluire, France). The instrument consists of a 3D Calvet calorimeter, which allows measuring the heat flow evolving during ACC transformation, and a symmetric microbalance, which is used to contemporaneously monitor the weight change of the sample. The relative humidity (RH = 85%) of the streaming gas was controlled by a humidity generator after equilibrating the samples at RH = 30% (*T* = 303 K). The calculation of the molar enthalpy of crystallization from the isothermal data is described in Text S3 in the Supporting Information.

*In Situ Total X‐Ray Scattering*: The powder samples were mounted on a cylindrical sample holder and placed inside a Linkam heating stage (Linkam FTIR600, Linkam Scientific Instruments, Surrey, UK). Two sets of in situ measurements were performed: (1) the stage temperature was increased from 25 to 400 °C at a rate of 2 °C min^−1^ and (2) the relative humidity was raised to a constant level of 85% and the temperature kept constant (*T* = 25 °C). X‐ray scattering patterns were acquired continuously at ID‐11 beamline at ESRF. Beam energy was set to 87 KeV, using a beam‐size with a 1 × 1 mm^2^ cross‐section. Data was acquired using a Frelon2K or a Frelon4M detector with a 10–30 s integration time. Data were processed using Fit2D software.^60^ Pair distribution functions were produced using the PDFgetX3 software.^61^

*Scanning Electron Microscopy*: Uncoated samples were investigated using a field emission scanning electron microscope (JEOL, JSM‐7500F) at acceleration energy of 5.0 keV.

## Conflict of Interest

The authors declare no conflict of interest.

## Supporting information

Supplementary
