# Supplementary material for: The Crystallization of Amorphous Calcium Carbonate is Kinetically Governed by Ion Impurities and Water
Source: Adv Sci (Weinh). 2018 Feb 14;5(5):1701000. doi: 10.1002/advs.201701000 (PMC5980180; doi:10.1002/advs.201701000)
Supplement: Supplementary file 1 — Supplementary [file ADVS-5-1701000-s001.pdf]

Copyright WILEY-VCH Verlag GmbH & Co. KGaA, 69469 Weinheim, Germany, 2018.

Supporting Information

for *Adv. Sci.,* DOI: 10.1002/advs.201701000

The Crystallization of Amorphous Calcium Carbonate is Kinetically Governed by Ion Impurities and Water

Marie Albéric, Luca Bertinetti, Zhaoyong Zou, Peter Fratzl, Wouter Habraken, and Yael Politi**

DOI: 10.1002/ advs.201701000
Article type: Full Paper

The crystallization of amorphous calcium carbonate is kinetically governed by ion impurities and water

Marie Albéric[Δ], Luca Bertinetti[Δ], Zhaoyong Zou, Peter Fratzl, Wouter Habraken and Yael Politi**

((Optional Dedication))

Dr. M. Albéric, Dr. L. Bertinetti, Dr. Z. Zou, Prof. P. Fratzl, Dr. W. Habraken and Dr. Y. Politi
Max-Planck Institute of Colloids and Interfaces Potsdam-Golm 14476, Germany
E-mail: yael.politi@mpikg.mpg.de, luca.bertinetti@mpikg.mpg.de

Supporting Information

The crystallization of amorphous calcium carbonate is kinetically governed by ion impurities and water

Marie Albéric[Δ], Luca Bertinetti[Δ], Zhaoyong Zou, Peter Fratzl, Wouter Habraken and Yael Politi**

			Atomic distances (Å)									
X-ray pair distribution function (PDF)	Samples	References (simulation method)	Ca-Oc	Ca-Ow	Oc-Oc	Oc-Ow	Oc-Oc	Ca-C	Ca-Ca	Ca-Oc	Ca-Ow	Ca-Ca
Partial PDF	Simulated ACC 1H_2O	Saharay et al. 2013 (MD)	2.4	2.4	-	-	-	2.9	3.9	4.1	4	6.1
	Simulated ACC 0.3H_2O		2.4	2.3	-	-	-	2.9	3.9	4.1	3.9	6.2
	Simulated ACC 1.3H_2O	Bushuev et al. 2015 (MD)	2.4	2.4	2.3	2.6	3	2.9	3.9	4.1	4	6
	Simulated ACC 0.5H_2O		2.4	2.4	2.3	2.6	2.9	2.9	3.9	4.1	4	6.1
Total PDF	Experimental ACC 1.3H_2O	Michel et al. 2008		2.4			2.9			4		6
	Simulated ACC 1H_2O	Saharay et al. 2013		2.4			3			-		-
	Simulated ACC 0.3H_2O			2.3			2.9			4.1		-
	Experimental ACC 1.4H_2O	Schmidt at al. 2014		2.4			2.9			4.1		6.2
	Experimental ACC 0.4H_2O			2.4			2.9			4.1		6.2
	Experimental ACC	Hydrated 1.4 H_2O		2.38			2.92			4.05		6.20
		Anhydrous 0.3 H_2O		2.36			2.94			4.02		6.24
		Crystalline		2.34			-			4.16		6.42
	Experimental Mg-ACC	Hydrated 1.4 H_2O		2.40			2.90			4.10		6.24
		Anhydrous 0.2 H_2O		2.37			2.90			4.08		6.24
		Crystalline		2.34			-			4.18		6.42
	Experimental P-ACC	Hydrated 1.3 H_2O		2.38			2.90			3.9 - 4.16		6.20
		Anhydrous 0 H_2O		2.36			2.92			4.00		6.24
		Crystalline		2.39			-			4.20		6.5

Table S1. Peak assignments of ACCs by comparison with the literature.

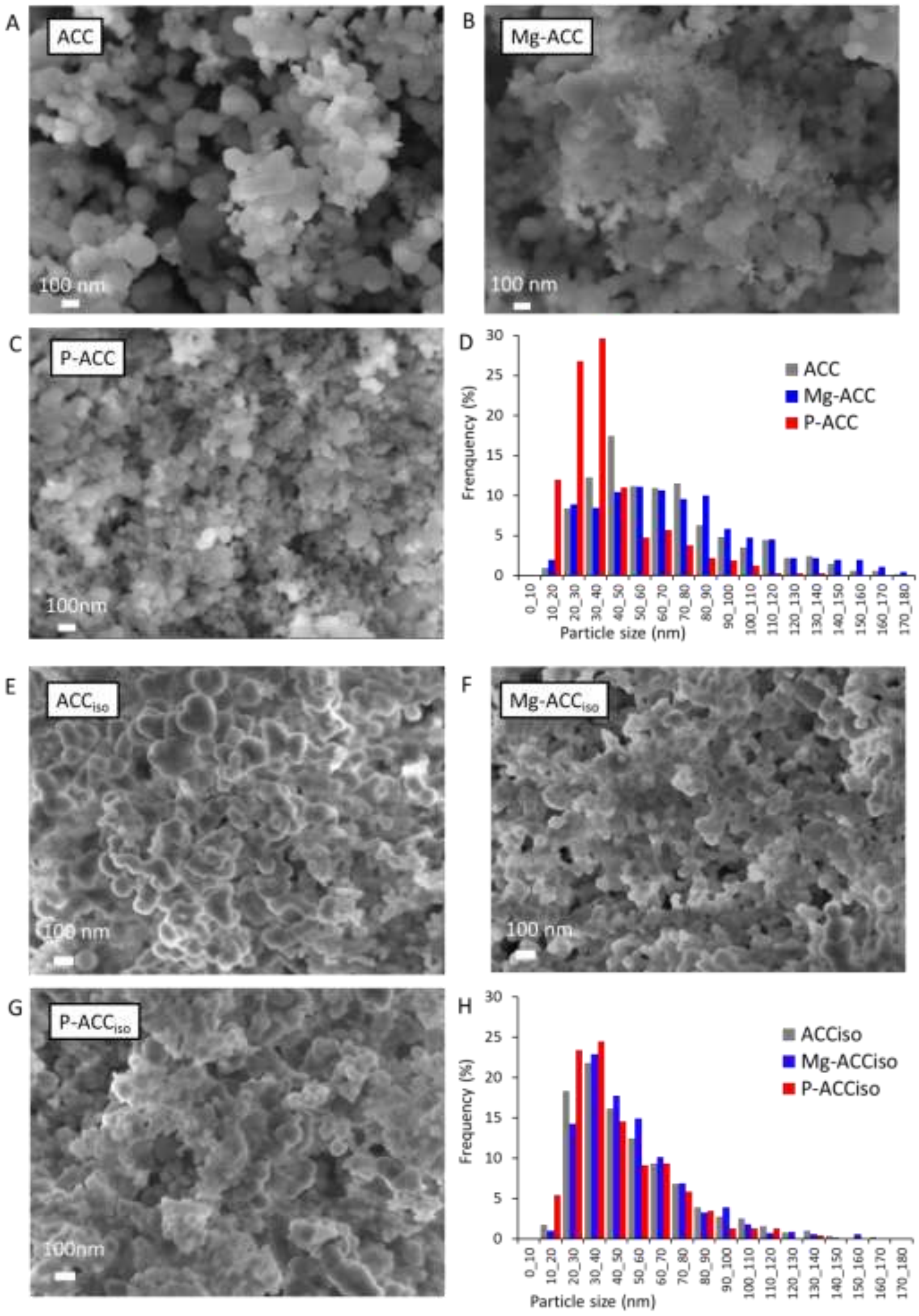

Figure S1. Scanning electron micrographs of synthetised ACCs. A) additive free ACC, B) Mg-ACC, C) P-ACC,D) comparison of the particle size distribution of ACC, Mg-ACC and P-ACC, E) additive free ACC_{iso}, F) Mg-ACC_{iso}, G) P-ACC_{iso},H) comparison of the particle size distribution of ACC_{iso}, Mg-ACC_{iso} and P-ACC_{iso}.

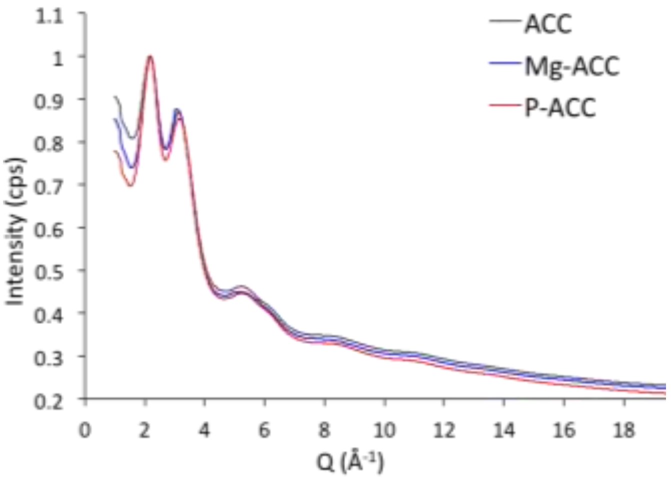

Figure S2. X-ray scattering profiles of hydrated additive free ACC, Mg-ACC and P-ACC (Normalized to the 1st maximum).

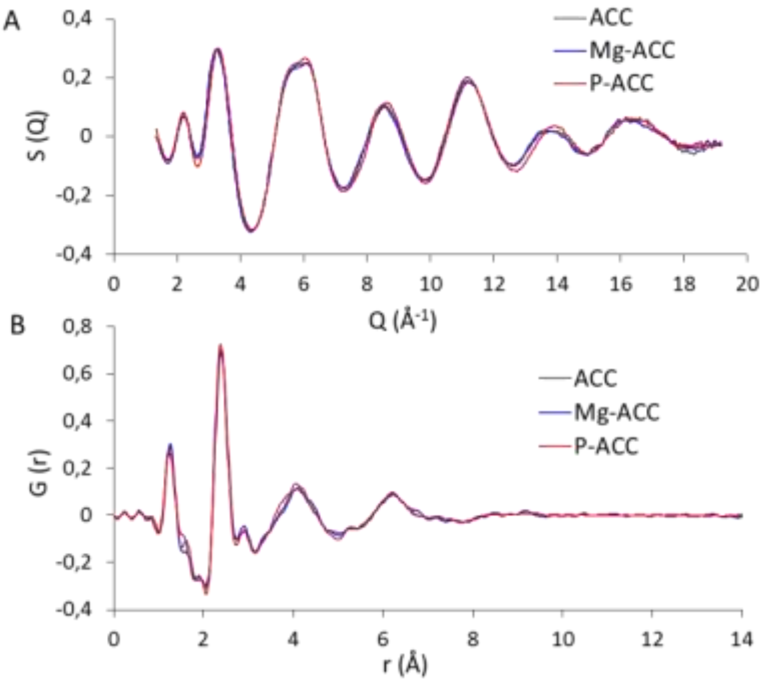

Figure S3. X-ray scattering results of ACCs. A) structure factor S(q) and B) calculated PDF G(r) as a function of r (Å).

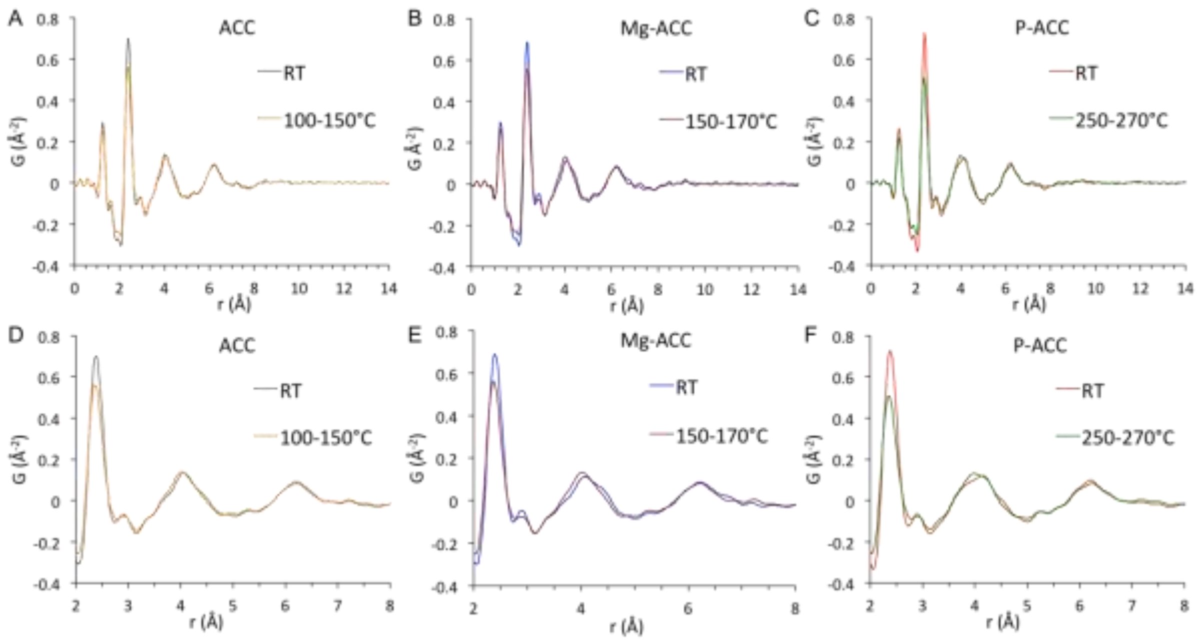

Figure S4. Computed pair distribution functions of hydrated and anhydrous samples**.** A) and D) starting ACC and ACC·0.4$H_2$0, C) and E) starting Mg-ACC with Mg-ACC·0.2 $H_2$0 and C) and F) starting P-ACC and P-ACC·0.01$H_2$0.

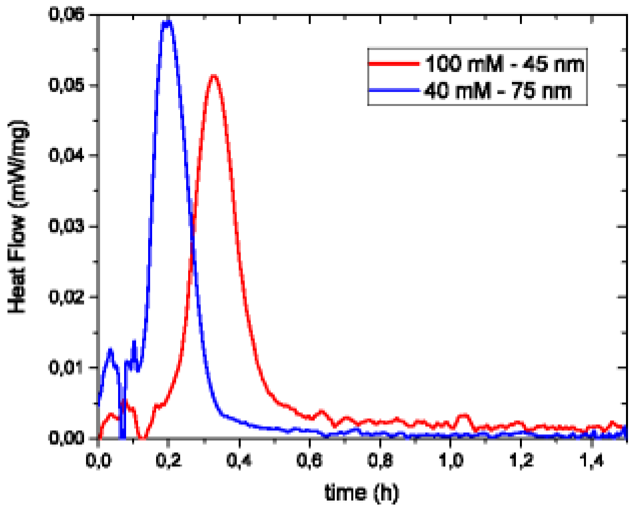

Figure S5. Effect of particle size on the kinetics of the humidity induced ACC crystallization. Crystallization heat flow for ACC·1H2O 100 mM (red) and 40 mM (blue), respectively with a particle size of around 45 nm and 75 nm.

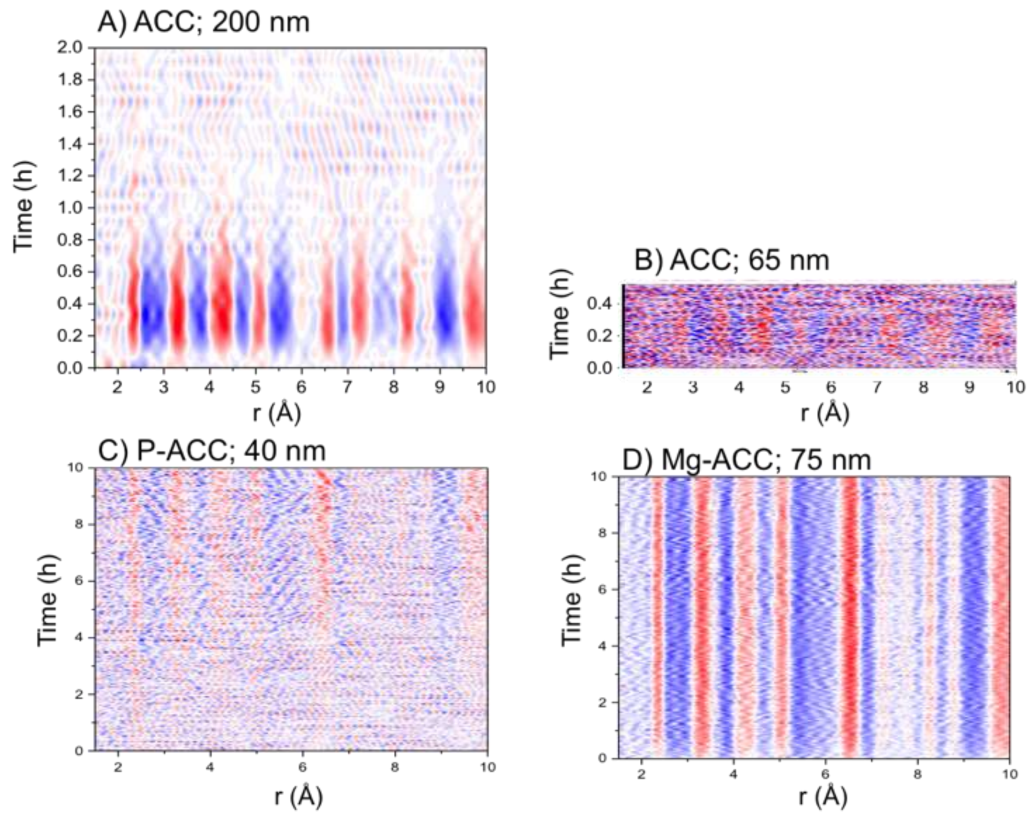

Figure S6. dPDF/dT maps calculated from x-ray in-situ humidity measurements. A) pure ACC with 200 nm particle size, B) 65 nm particle size, C) P-ACC; 40 nm particle size and D) Mg- ACC; 75 nm particle size.

Text S1. Surface water calculation

The amount of water determined in this work refers mainly to bulk water. For the TG experiments, in fact, the samples were equilibrated for a relatively long time at RT under dry N2. For this reason, most of the surface water was already removed. The residual surface water could be estimated as follows: considering particles of 80 nm in diameter and a density for hydrated ACC of about 2.3 g/cm^3, the specific surface area of ACC would be about 30-35 m^2/g. Assuming (overestimating) that the water size is 2.8 Å and that a full monolayer is left after dehydration at RT, we can assess the surface water contribution to be maximally about 10% of the total water. More realistically, only the strongly bound water is left at the surface of ACC after equilibration at low RHs, which has been estimated to be about 3 wat/nm^2. [42] In this case, the contribution to the surface water here would represent maximally 2% of the total water content.

Text S2. Calculation of the entropic contribution -TdΔS/dn to the molar free energy

The experimentally standard entropy values of calcite and hydrated calcium carbonates, namely monohydrocalcite ($CaCO_3 \cdot H_2O$) and ikaite ($CaCO_3 \cdot 6H_2O$) were used to calculate the entropic contribution -TdΔS/ dn_{H2O} to the molar free energy. The entropy of calcite $S_{calcite}$ was reported to vary between 91.7 and 92.9 J K^{-1} mol^{-1}, [50] [51] the entropy of monohydrocalcite S_{mhc} to be 129.7 J $K^{-1}mol^{-1}$[52] [53] and the one of ikaite S_{ikaite} to range from 306.6 [62] to 310.40 J K^{-1} mol^{-1} [53]. Therefore, -TdΔS/ dn_{H2O} for the hydration of calcite to monohydrocalcite at for example 298K is (S_{mhc} - $S_{calcite}$)*298/1=10.6-10.9 kJ mol^{-1} and for the hydration of calcite to ikaite (S_{ikaite} - $S_{calcite}$)*298/6=10.9- 11.3 kJ mol^{-1}. For Figure 2C an average of 10.9 kJ mol^{-1} with a standard deviation of 0.3 was used for -TdΔS/ dn_{H2O}. Note that –dΔS/ dn_{H2O} is 0.036 kJ mol^{-1}.

Text S3 Microgravimetry and isothermal calorimetry.

As the starting material is hydrated, the transformation is accompanied by a weight loss due to water evaporation. The total heat flow measured over time, Q(t) [Js^{-1}], then consists of both a (exothermic) contribution due to the ACC crystallization, $Q_c(t)$, and a (endothermic) contribution associated to water vaporization $Q_v(t)$. To extract the crystallization contribution, the measurement of the heat flow is subtracted for the water vaporization contribution, i.e. $Q_c(t)=Q(t)-Q_v(t)$. $Q_v(t)$ is determined by multiplying the rate of weight change m'(t)=dm/dt [gs^{-1}] by the specific enthalpy of vaporization of water $\Delta_v H$ [Jg^{-1}]. The molar enthalpy of crystallization at 30 °C, $\Delta_c H^{30}$, can then be obtained by integration of $Q_c(t)$ over the transformation time:

$$\Delta_c H^{30} = -\frac{1}{n}\int_{t_0}^{t_f} Q_c(t)dt = -\frac{1}{n}\int_{t_0}^{t_f} Q(t) - \Delta_v H \cdot m'(t)dt$$

where t_0 and t_f are respectively the time at which the crystallization starts and the time at which the process is completed and n_c is the number of moles of calcium carbonates calculated from the mass of the sample at t_f. To accurately subtract the vaporization contribution and to determine the error on the heat flow measurements in isothermal conditions, we measured $\Delta_v H$ directly in our setup by placing around 40 mg of water in an aluminium crucible and increasing step-wise the RH of the streaming gas. At constant RH the evaporation rate is constant and depends on the activity of water in the streaming gas. This results in a step-wise change in the heat flow which can be fitted using the equation: $Q_v(t)= \Delta_v H\ m'(t) + Q_0$. Using $\Delta_v H$ as a fitting parameter and the calorimeter baseline Q_0, we obtained $\Delta_v H$=43.1 kJ mol^{-1} at 30°C, which is very close to the reported enthalpy of vaporization of water (44 kJ mol^{-1}). This allows assessing the accuracy of the setup in isothermal conditions to be about 1%. The value of 43.1 kJ mol^{-1} was then used to subtract for the contribution of the vaporization.

Other thermal events, namely, water adsorption and dissolution, may contribute to the total heat flow. We considered these contributions that are however expected to be negligible.

Water adsorption:

For the transformation induced by humidity, as indicated in the SI, samples were equilibrated at a water vapour pressure P/P0 of 0.3. As typical for Calcium salts, at that P/P0 enthalpy of adsorption are not larger than 45-46 kJ/mol[63]. This is in fact the reason why we chose to equilibrate the samples at a relatively high P/P_0. Unfortunately, there are no such reference data for ACC. If only water with partial enthalpy of adsorption very close to 44 kJ/mol is adsorbed, the measurement is only very slightly (if at all) affected by this phenomenon. It may have been unclear from our text that by simultaneously measuring the weight of the sample, we can compensate not only water evaporation, but also for the condensation of water from gas phase during sorption. After changing the relative humidity from 30% to 85%, therefore, the contribution to the heat of adsorption of water will be maximally 1-2 kJ/mol of water.

Taking an average value of 80 nm for particle diameter, we can easily estimate that the maximum heat produced by adsorption for a full monolayer of water is smaller than 2 J/g of CaCO3. The heat of crystallisation, on the other hand, is, in the worst case (i.e. for the most hydrated samples), around 50 J/g of CaCO3. Therefore, the contribution of water adsorption does not exceed 1-4% of the total measured enthalpy changes. We have added this consideration in the description of the method in the SI.

In addition to these theoretical, overestimated, values, we can show experimentally that water sorption does not have a significant contribution to the crystallization heat flow. This is especially clear in the heat flow trace of those ACCs which are partially dehydrated and where the crystallisation occurs at later time points. Whereas the contribution of water sorption should result in a peak lasting few minutes right after the RH change step, no significant net HF peak can be observed in the case of these samples within that timeframe.

The same can be observed in Fig. S7: the crystallisation of ACC starts after around 3h (i.e. around 20 min after the RH change, see orange trace in panel B) but the initial adsorption of water (witnessed by the initial weight increase in the green trace in panel A) is almost over once ACC starts transforming. Clearly, the calculated heat of adsorption/desorption from gas phase (green trace in panel B) corrects the measured heat flow (blue trace in panel B) for this effect very well, as there is no net peak between 0 and 20 minutes after humidity change.

Therefore, we can confidently conclude that our values for the crystallization enthalpy are not

Dissolution:

We do not exclude that the sample undergoes dissolution and re-precipitation. In fact, we have suggested that this is the mechanism for the transformation at high humidity levels based on our PDF analyses. Nevertheless, we are measuring the sum of both processes, not just one of the two. This means that, independently of the transformation mechanisms, and assuming that the transformation proceeds completely (i.e. there is no ACC left), we are measuring the difference between the initial and the final states, i.e. $ACC\cdot nH_2O$ and calcite $+nH_2O$. This is

the equivalent of evaluating the enthalpy changes following the Hess' law of constant heat summation with the following reaction steps:

ACC*nH_2O(s) → Ca^{2+}(aq) + CO_3^{2-}(aq) + nH_2O(l)   ΔH1

Ca^{2+}(aq) + CO_3^{2-}(aq) + nH_2O(l) → calcite (s) + nH_2O (l)   ΔH2

The ΔH for the reaction

ACC·nH2O(s) → calcite (s) + n H2O (l)

is then equal to ΔH1+ΔH2.

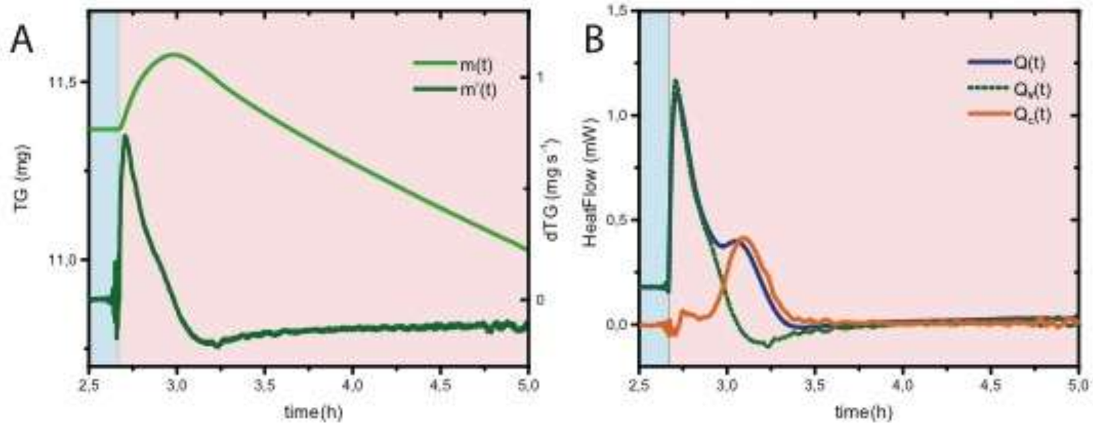

Figure S7. Measure of the crystallization enthalpy for ACC. A) The sample is equilibrated at 303 K at a RH of 30% (blue shadowed area) until its weight (m(t), light green) is constant. Then the humidity is changed to 85% (red shadowed area) to induce the crystallization. After a first weight increase due to water adsorption at the surface of the particles, the material starts crystallizing and a weight loss is observed. The rate of weight change (m'(t), dark green) is easily calculated from the instantaneous weight. B) The heat flow contribution due to crystallization (Q_c(t), orange) is obtained from the total heat flow (Q(t), blue) by subtraction of the heat flow due to water vaporization (Q_v(t), dashed green) calculated from the rate of weight change.

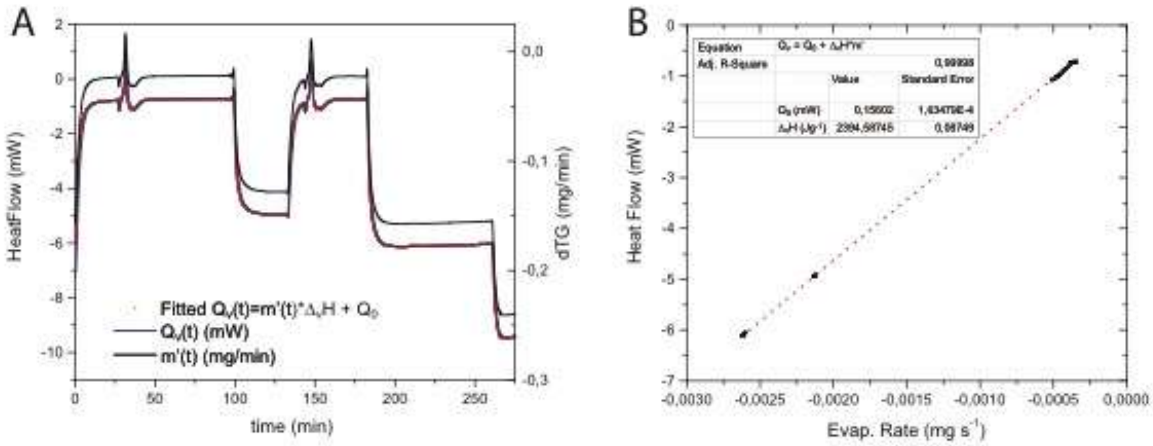

Figure S8. Validation of the accuracy of the calorimeter. A) About 40 mg of water are placed in a crucible inside the calorimeter. The humidity is changed stepwise and the heat flow (Q_v(t), blue) as well as the rate of weight change (m'(t), black) are monitored overtime. B) Plotting the linear parts of the curves against each other, the enthalpy of evaporation of water $\Delta_v H$ as well as the calorimeter baseline Q_0 can be evaluated with a simple linear fit. The obtained value for the enthalpy of vaporization at 303 K. C is about 43.1 kJ mol-1, which is about 1.5% lower than 43.7 kJ mol^{-1} reported in literature.

References

[1] E. Beniash, J. Aizenberg, L. Addadi, S. Weiner, P Roy Soc B-Biol Sci **1997**, *264*, 461.
[2] S. Weiner, L. Addadi, in *Annual Review of Materials Research, Vol 41*, Vol. 41 (Eds: D. R. Clarke, P. Fratzl), Annual Reviews, Palo Alto 2011, 21.
[3] Y. Politi, J. C. Weaver, Science **2015**, *347*, 712.
[4] S. Sviben, A. Gal, M. A. Hood, L. Bertinetti, Y. Politi, M. Bennet, P. Krishnamoorthy, A. Schertel, R. Wirth, A. Sorrentino, E. Pereiro, D. Faivre, A. Scheffel, Nat Commun **2016**, *7*, 9.
[5] T. Mass, A. J. Giuffre, C. Y. Sun, C. A. Stifler, M. J. Frazier, M. Neder, N. Tamura, C. V. Stan, M. A. Marcus, P. Gilbert, P Natl Acad Sci USA **2017**, *114*, E7670.
[6] K. Lee, W. Wagermaier, A. Masic, K. P. Kommareddy, M. Bennet, I. Manjubala, S. W. Lee, S. B. Park, H. Colfen, P. Fratzl, Nat Commun **2012**, *3*, 7.
[7] V. Reichel, A. Kovacs, M. Kumari, E. Bereczk-Tompa, E. Schneck, P. Diehle, M. Posfai, A. M. Hirt, M. Duchamp, R. E. Dunin-Borkowski, D. Faivre, Sci Rep **2017**, *7*, 8.
[8] Y. Oaki, Bull. Chem. Soc. Jpn. **2017**, *90*, 776.
[9] L. Addadi, S. Raz, S. Weiner, Adv Mater **2003**, *15*, 959.
[10] J. J. De Yoreo, P. Gilbert, N. Sommerdijk, R. L. Penn, S. Whitelam, D. Joester, H. Z. Zhang, J. D. Rimer, A. Navrotsky, J. F. Banfield, A. F. Wallace, F. M. Michel, F. C. Meldrum, H. Colfen, P. M. Dove, Science **2015**, *349*.
[11] Y. Levi-Kalisman, S. Raz, S. Weiner, L. Addadi, I. Sagi, Adv Funct Mater **2002**, *12*, 43.
[12] S. Raz, P. C. Hamilton, F. H. Wilt, S. Weiner, L. Addadi, Adv Funct Mater **2003**, *13*, 480.
[13] Y. Politi, D. R. Batchelor, P. Zaslansky, B. F. Chmelka, J. C. Weaver, I. Sagi, S. Weiner, L. Addadi, Chem Mater **2010**, *22*, 161.
[14] A. Al-Sawalmih, C. H. Li, S. Siegel, P. Fratzl, O. Paris, Adv Mater **2009**, *21*, 4011.
[15] J. Ihli, W. C. Wong, E. H. Noel, Y. Y. Kim, A. N. Kulak, H. K. Christenson, M. J. Duer, F. C. Meldrum, Nat Commun **2014**, *5*.
[16] A. L. Goodwin, F. M. Michel, B. L. Phillips, D. A. Keen, M. T. Dove, R. J. Reeder, Chem Mater **2010**, *22*, 3197.
[17] F. M. Michel, J. MacDonald, J. Feng, B. L. Phillips, L. Ehm, C. Tarabrella, J. B. Parise, R. J. Reeder, Chem Mater **2008**, *20*, 4720.
[18] A. V. Radha, A. Fernandez-Martinez, Y. D. Hu, Y. S. Jun, G. A. Waychunas, A. Navrotsky, Geochim Cosmochim Ac **2012**, *90*, 83.
[19] R. J. Reeder, Y. Z. Tang, M. P. Schmidt, L. M. Kubista, D. F. Cowan, B. L. Phillips, Cryst Growth Des **2013**, *13*, 1905.
[20] A. Becker, U. Bismayer, M. Epple, H. Fabritius, B. Hasse, J. M. Shi, A. Ziegler, Dalton T **2003**, 551.

[21] B. Hasse, H. Ehrenberg, J. C. Marxen, W. Becker, M. Epple, Chem-Eur J **2000**, *6*, 3679.
[22] Y. Politi, Y. Levi-Kalisman, S. Raz, F. Wilt, L. Addadi, S. Weiner, I. Sagi, Adv Funct Mater **2006**, *16*, 1289.
[23] J. C. Marxen, W. Becker, D. Finke, B. Hasse, M. Epple, J Mollus Stud **2003**, *69*, 113.
[24] C. C. Tester, C. H. Wu, M. R. Krejci, L. Mueller, A. Park, B. Lai, S. Chen, C. J. Sun, M. Balasubramanian, D. Joester, Adv Funct Mater **2013**, *23*, 4185.
[25] P. Rez, S. Sinha, A. Gal, J Appl Crystallogr **2014**, *47*, 1651.
[26] Y. Politi, R. A. Metzler, M. Abrecht, B. Gilbert, F. H. Wilt, I. Sagi, L. Addadi, S. Weiner, P. U. P. A. Gilbert, P Natl Acad Sci USA **2008**, *105*, 17362.
[27] Y. U. T. Gong, C. E. Killian, I. C. Olson, N. P. Appathurai, A. L. Amasino, M. C. Martin, L. J. Holt, F. H. Wilt, P. U. P. A. Gilbert, P Natl Acad Sci USA **2012**, *109*, 6088.
[28] R. T. DeVol, C. Y. Sun, M. A. Marcus, S. N. Coppersmith, S. C. B. Myneni, P. Gilbert, J Am Chem Soc **2015**, *137*, 13325.
[29] A. V. Radha, T. Z. Forbes, C. E. Killian, P. U. P. A. Gilbert, A. Navrotsky, P Natl Acad Sci USA **2010**, *107*, 16438.
[30] A. Gal, K. Kahil, N. Vidavsky, R. T. DeVol, P. Gilbert, P. Fratzl, S. Weiner, L. Addadi, Adv Funct Mater **2014**, *24*, 5420.
[31] S. Kababya, A. Gal, K. Kahil, S. Weiner, L. Addadi, A. Schmidt, J Am Chem Soc **2015**, *137*, 990.
[32] Z. Y. Zou, L. Bertinetti, Y. Politi, A. C. S. Jensen, S. Weiner, L. Addadi, P. Fratzl, W. J. E. M. Habraken, Chem Mater **2015**, *27*, 4237.
[33] C. Rodriguez-Navarro, K. Kudlacz, O. Cizer, E. Ruiz-Agudo, Crystengcomm **2015**, *17*, 58.
[34] D. J. Tobler, J. D. R. Blanco, H. O. Sorensen, S. L. S. Stipp, K. Dideriksen, Cryst Growth Des **2016**, *16*, 4500.
[35] F. Konrad, F. Gallien, D. E. Gerard, M. Dietzel, Cryst Growth Des **2016**, *16*, 6310.
[36] S. Sen, D. C. Kaseman, B. Colas, D. E. Jacob, S. M. Clark, Phys. Chem. Chem. Phys. **2016**, *18*, 20330.
[37] M. F. Khouzani, D. M. Chevrier, P. Guttlein, K. Hauser, P. Zhang, N. Hedin, D. Gebauer, Crystengcomm **2015**, *17*, 4842.
[38] W. J. E. M. Habraken, A. Masic, L. Bertinetti, A. Al-Sawalmih, L. Glazer, S. Bentov, P. Fratzl, A. Sagi, B. Aichmayer, A. Berman, J Struct Biol **2015**, *189*, 28.
[39] Y. G. Bushuev, A. R. Finney, P. M. Rodger, Cryst Growth Des **2015**, *15*, 5269.
[40] M. Saharay, A. O. Yazaydin, R. J. Kirkpatrick, J Phys Chem B **2013**, *117*, 3328.
[41] M. P. Schmidt, A. J. Ilott, B. L. Phillips, R. J. Reeder, Cryst Growth Des **2014**, *14*, 938.
[42] A. V. Radha, A. Navrotsky, Cryst Growth Des **2015**, *15*, 70.
[43] S. Y. Yang, H. H. Chang, C. J. Lin, S. J. Huang, J. C. C. Chan, Chemical Communications **2016**, *52*, 11527.
[44] G. Cobourne, G. Mountjoy, J. D. Rodriguez-Blanco, L. G. Benning, A. C. Hannon, J. R. Plaisier, J Non-Cryst Solids **2014**, *401*, 154.
[45] D. Gebauer, P. N. Gunawidjaja, J. Y. P. Ko, Z. Bacsik, B. Aziz, L. J. Liu, Y. F. Hu, L. Bergstrom, C. W. Tai, T. K. Sham, M. Eden, N. Hedin, Angew Chem Int Edit **2010**, *49*, 8889.
[46] C. J. Lin, S. Y. Yang, S. J. Huang, J. C. C. Chan, J Phys Chem C **2015**, *119*, 7225.
[47] A. Navrotsky, C. Capobianco, Am Mineral **1987**, *72*, 782.
[48] N. Koga, Y. Z. Nakagoe, H. Tanaka, Thermochimica Acta **1998**, *318*, 239.
[49] A. Navrotsky, P Natl Acad Sci USA **2004**, *101*, 12096.
[50] L. A. K. Staveley, R. G. Linford, The Journal of Chemical Thermodynamics **1969**, *1*, 1.
[51] D. V. Schroeder, *An Introduction to Thermal Physics*, Pearson Education (US), San Francisco, United States 1999.

[52] H. Hull, A. G. Turnbull, Geochim Cosmochim Ac **1973**, *37*, 685.
[53] E. Königsberger, L.-C. Königsberger, H. Gamsjäger, Geochim Cosmochim Ac **1999**, *63*, 3105.
[54] P. Raiteri, J. D. Gale, J Am Chem Soc **2010**, *132*, 17623.
[55] S. Raz, O. Testeniere, A. Hecker, S. Weiner, G. Luquet, Biological Bulletin **2002**, *203*, 269.
[56] K. Okazaki, S. Inoue, Dev. Growth Diff. **1976**, *18*, 413.
[57] C. C. Tester, M. L. Whittaker, D. Joester, Chemical Communications **2014**, *50*, 5619.
[58] L. J. de Nooijer, T. Toyofuku, H. Kitazato, P Natl Acad Sci USA **2009**, *106*, 15374.
[59] Y. Politi, J. Mahamid, H. Goldberg, S. Weiner, L. Addadi, Crystengcomm **2007**, *9*, 1171.
[60] A. P. Hammersley, S. O. Svensson, M. Hanfland, A. N. Fitch, D. Hausermann, High Pressure Res **1996**, *14*, 235.
[61] P. Juhas, T. Davis, C. L. Farrow, S. J. L. Billinge, J Appl Crystallogr **2013**, *46*, 560.
[62] J. L. Bischoff, J. A. Fitzpatrick, R. J. Rosenbauer, J. Geol. **1993**, *101*, 21.
[63] V. Bolis, C. Busco, G. Martra, L. Bertinetti, Y. Sakhno, P. Ugliengo, F. Chiatti, M. Corno, N. Roveri, Philos. Trans. R. Soc. A-Math. Phys. Eng. Sci. **2012**, *370*, 1313.
